# Determination of Chemical Composition and Antimicrobial Activity of the CO_2_ Extract of *Eryngium planum* L.

**DOI:** 10.1155/2023/4702607

**Published:** 2023-04-27

**Authors:** Aliya B. Arykbayeva, Gulbaram O. Ustenova, Kamalidin O. Sharipov, Ulzhan T. Beissebayeva, Irina E. Kaukhova, Auyes Myrzabayeva, Nadezhda G. Gemejiyeva

**Affiliations:** ^1^Department of Pharmaceutical Technology, Asfendiyarov Kazakh National Medical University, Almaty 050000, Kazakhstan; ^2^Department of Biochemistry, Asfendiyarov Kazakh National Medical University, Almaty 050000, Kazakhstan; ^3^Department of Dermatovenereology, Asfendiyarov Kazakh National Medical University, Almaty 050000, Kazakhstan; ^4^Department of Industrial Technology of Medicines, St. Petersburg State Chemical Pharmaceutical University, Saint Petersburg 197376, Russia; ^5^Scientific Center for Anti-Infectious Drugs JSC, Almaty 050000, Kazakhstan; ^6^Laboratory of Plant Resources, Institute of Botany and Phyto-Introductions, Almaty 050040, Kazakhstan

## Abstract

The article presents parameters for obtaining a carbon dioxide extract from the subterranean part of *Eryngium planum* that contains a valuable set of organic substances and has a certain antimicrobial effect. *Methods*. Raw materials were collected in the Almaty region (Republic of Kazakhstan). The CO_2_ extract of *Eryngium planum* herbs was obtained under subcritical conditions. A gas chromatograph with a mass spectrometric detector was used to determine the compositional breakdown of the extract. Antimicrobial activity was determined by two methods: the micromethod of serial dilutions and the disk-diffusion method. Three microbial test strains were used: *Staphylococcus aureus* ATCC 6538-P, *Escherichia coli* ATCC 8739, and *Candida albicans* ATCC 10231. *Results*. To extract biologically active substances from the subterranean part of *Eryngium planum* L., we have chosen carbon dioxide extraction technology, a technology for processing carbon dioxide (CO_2_) raw materials, which allows us to extract various substances in high concentrations. Carbon dioxide extraction technology is an effective and environmentally safe way to isolate various biologically active substances contained in medicinal plant raw materials. In the composition of the CO_2_ extract of *Eryngium planum* L. 43 components were identified, the main of which are *α*-linolenic acid, 8.30%; myristic acid, 6.40%; caryophyllene, 6.92%; spatulous, 6.62%; and other main identified compounds and their percentage. *Conclusions*. The study showed that the CO_2_ extract of *Eryngium planum* L. contains biologically active compounds that have a pronounced antimicrobial effect against clinically significant microorganisms, such as *Escherichia coli, Staphylococcus aureus,* and *Candida albicans*.

## 1. Introduction

A source of raw materials for the production of effective and safe medicines has been increasing as a late interest of scientific medicine in medicinal plants. Preparations made of plant raw materials are currently widely used for the treatment and prevention of many diseases [1]. Their assortment is expanding, and the number of phytopreparations is increasing annually [2]. The advantages of phytopreparations over synthetic drugs are their mild action and low toxicity.

The flora of Kazakhstan has great potential as a source of promising medicinal forms. It is known that many promising plant species for medicine are poorly studied from the standpoint of botanical resource studies and pharmacogenetic analysis [3]. Such plants also include species of *Eryngium*.

The *Apiaceae* family includes about 450 genera and 3700 species worldwide [3, 4]. The plants of this family are well known as medicinal plants. The members of the *Apiaceae* possess various compounds with many biological activities [4].

Representatives of the genus *Eryngium* L. are found in tropical, subtropical, and temperate zones, mainly in Mexico and South America. They grow in sandy places, thickets of shrubs, and steppes [5].

Some species, such as *E. foetidum* L., *E. maritime* L., *E. campestris* L., and *E. creticum* Lam. are used in ethnomedicine all over the world [6]. *Eryngium foetidum* L. has a strong aroma and contains essential oil, which is valuable for pharmaceutical, perfume, and flavor industries [7].

It is known that foreign scientific papers have conducted studies on the chemical composition and pharmacological activity of such representatives of the genus: *Eryngium planum, E. aquaticum, E. foetidum, E. alpinum, E. campestris, E. amethystine, E. triquetrum, E. caucasicum, E. barrelieri, E. glomeratum, E. kitschy, E. maritime,* and *E. coeruleus* [8–11]. Essential oils, polyacetylenes, lignans, phenols, and flavonoids were isolated from the raw materials of these plants. Extracts of these plants have following properties: cytotoxic, antibacterial, antimicrobial, anti-inflammatory, diuretic, antiallergic, and antifungal [12–15].

There are some species of bluebirds in our country such as *Eryngium caucasicum* Trout., *Eryngium macro calyx Shrink, and Eryngium planum* L.

*Eryngium planum* is perennial and found in the steppes of northern Kazakhstan and the mountains of Dzungarian and Trans-Ili Alatau [3]. The plant is 30–90 cm tall. All parts of the plant, especially the upper part, have blue or purple hues. It has a straight taproot; the stem is bluish, and in the upper part, it is branched. The leaves are leathery, with prickly teeth along the edge, and the lower leaves are petiolate and oval, whole up is to 15 cm long. The leaves, which are obscure in the middle part of the stem, are on shorter petioles. The upper leaves are palmately divided into 3–5 sessile lobes.

The flowers are collected in dense ovoid heads (up to 2 cm long). The petals are bluish. Wrapper sheets with prickly teeth. It blooms from June to July and begins to bloom in the second year of life. The fruit is ovoid and covered with scales [16, 17].

*Eryngium planum* is found in clearings, meadows, forest edges, on sand, along river banks, and along roads in Kazakhstan.

The general distribution occurs in Central and Southern Europe, Eastern Europe, the Caucasus, Eastern Transcaucasia, Central Asia, and Russia [18].

According to the literature, *Eryngium planum* contains essential and fatty oils, carbohydrates, organic and phenolic carboxylic acids, triterpenoids, polyacetylene compounds, coumarins, flavonoids, saponins, and vitamin C. It is used as an antispasmodic, diuretic, sedative, detoxifying, and anti-inflammatory agent [19–21]. According to literature data, triterpene saponins are the main active substances responsible for expectorant, depurative, antioxidative, and diuretic effects [21].

The authors in [22] studied the composition of the essential oil of *Eryngium planum*. The analysis of the composition of essential oils showed the oil from different parts of *Eryngium planum* in vivo, and also, in vitro sprouts can be a source of falcarinol and polyacetylenes, which are important compounds that promote health. Falcarinol is a biologically active compound in the group of falcarinol-type polyacetylenes. It shows pronounced cytotoxic activity against human tumor cells in vitro and also has antitumor activity in vivo.

Suciu and Pârvu evaluated the effect of liquid extract (ethanol 70%) of *Eryngium planum* to reduce acute inflammation caused by turpentine oil in rats. The tested extract showed an anti-inflammatory effect due to a decrease in the total number of leukocytes and neutrophil distribution and activity [23].

Oxidative stress plays an important role in the development and progression of many diseases, including neurodegenerative diseases [24]. The results of epidemiological studies indicate that higher intake of antioxidant compounds is associated with a lower risk of mortality from cancer and coronary heart disease [25].

Studies show the antioxidant potential of methanol and methanol-water extracts of two plant species *Eryngium amethystine* and *Eryngium planum* using beta-carotene-linolenic acid analysis. The results show that the water-methanol extracts of *amethystine* and *planum* are more powerful in terms of antioxidant content than methanol extracts. It was also found that *plant* extracts exhibit a stronger antioxidant effect than similar extracts for *E. amethystine* [25].

Thus, extracts of the subterranean part of *Eryngium planum* L. have a wide range of pharmacological properties due to the content of various groups of biologically active compounds. Therefore, *Eryngium planum* L. is very promising for further phytochemical study of its composition and therapeutic effects.

Currently, the technology of extraction of vegetable raw materials with compressed and liquefied gases has been proposed and is actively developing. Liquefied CO_2_ is used to isolate essential and fatty hydrophobic substances. Hydrophilic substances are well extracted by liquefied gas, having high dielectric conductivity. When extracted with liquefied gas, the extractant evaporates in a high-pressure stream and extractive substances remain in pure form [26, 27]. The tendency to use liquefied and compressed gases and, in particular, carbon dioxide for these purposes, the extraction process can be carried out under pre- and supercritical conditions [27].

Supercritical fluid extraction with carbon dioxide has some minor disadvantages. One of them is the extraction plant's high engineering requirements and investment costs due to high pressure requirements.

Advantages of CO_2_-extraction under subcritical conditions compared to supercritical are primarily due to the fact that this process is highly cost-effective, more technologically advanced, allows processing not only high-quality raw materials, but also production waste in order to extract the main components from them [28, 29].

In this study, we obtained a thick CO_2_ extract under subcritical conditions from the subterranean part of *Eryngium planum* and studied the compositional breakdown and its antimicrobial activity against pathogenic microorganisms for the first time.

## 2. Materials and Methods

### 2.1. Plant Material

The subterranean part of *Eryngium planum* was the chosen study material collected from July to August during the flowering phase in the Sogeti Mountains, on the territory of the Enbekshi Kazakh district of the Almaty region (coordinates: *H* = 1086 m above sea level, *N* = 43°27′05.8″, and *E* = 078°39′12.7″). The plant was identified by the Institute of Botany and Phytointroduction of the Forestry and Wildlife Committee of the Ministry of Ecology, Geology, and Natural Resources of the Republic of Kazakhstan.

The collected vegetable raw materials were naturally dried in the shade and in a well-ventilated room at a temperature of +25 ± 5°. The moisture content of vegetable raw materials did not exceed 10%. The dried vegetable raw materials were crushed using a KDU-2 crusher. The particle size of vegetable raw materials was 1–3 mm.

The extraction mass of medicinal plant raw materials was 1800 g, of which 30 g of extract was obtained; the yield was 1.67%. In appearance, a thick CO_2_ extract obtained under subcritical conditions from *Eryngium planum* has a brown color and a specific smell.

### 2.2. Preparation of Carbon Dioxide Extract

The dried subterranean part of *Eryngium planum* was used as a medicinal plant raw material for the production of the carbon dioxide extract. The extract was obtained at the production base of Zhanafarm Medicine Production LLP. The extract was obtained under subcritical conditions on an extraction unit with a volume of 5 liters in accordance with the standard of the enterprise. Liquid carbon dioxide was used as an extractant. Optimal conditions for obtaining the CO_2_ extract were maintained as follows: pressure was 40–51 atm, temperature was 18–21°C, and the extraction time was 11 hours.

### 2.3. Determination of the Compositional Breakdown of the Extract

The compositional breakdown of the extract for the content of organic substances was determined using a gas chromatograph with a mass spectrometric detector (Agilent 7890A/5975C).

Chromatography conditions were as follows: sample volume 0.2 *μ*l and sample inlet temperature 240°C, without flow separation. Separation was carried out using a capillary chromatographic column DB-35MS (Agilent, USA), with a length of 30 m, an inner diameter of 0.25 mm, and a film thickness of 0.25 microns at a constant velocity of carrier gas (helium) 1 ml/min. The chromatography temperature was programmed from 50°C (1 min exposure) to 270°C at a heating rate of 10°C/min (15 min exposure). Detection was carried out in the SCAN mode *m*/*z* 34–750. Agilent MSD Chem Station software was used to control the gas chromatography system and record and process results and data. The Wiley 7th edition and NIST′02 data libraries were used to identify the obtained mass spectra. Data processing included determination of retention times and peak areas, and verification of spectral information was obtained using a mass spectrometric detector.

### 2.4. Determination of Antimicrobial Activity

The antimicrobial activity of the carbon dioxide extract of *Eryngium planum* L. was determined by two methods: the serial dilution method and the disk-diffusion method [30–32].

To study antimicrobial activity, standard test strains of microorganisms were used as follows: *Staphylococcus aureus* ATCC 6538-P obtained from the Republican Collection of Microorganisms (Nur-Sultan, Kazakhstan) and *Candida albicans* ATCC 10231 and *Escherichia coli* ATCC 8739 obtained from the American Collection of Type cultures (ATCC, USA).

Sensitivity studies on microorganisms were carried out on standard nutrient media: Muller–Hinton agar (M173), HI Media, India; Muller–Hinton broth (Muller–Hinton broth (M391), HI Media, India (CLSI); and Sabouraud liquid medium (M013), HI Media, India (CLSI).

#### 2.4.1. Antimicrobial Activity Assay of the Extract by the Serial Dilution Method

A 96-well plate was used to determine antimicrobial activity. Muller–Hinton nutrient broth (M391) was added to all wells for testing bacteria, and Sabouraud broth was used for testing mushrooms in the amount of 150 ml (from the 1st to the 24th well). The extract was introduced into the 1st well at a base concentration of 150 *μ*l, and serial dilutions were carried out, which were prepared using a mixture of Muller–Hinton broth/Saburo dextrose broth (150 *μ*l) + test sample (150 ml) from the 1st well. a test tube in the amount of 150 ml into the 2nd test tube, already containing 150 ml of broth. 150 ml of the test sample in the broth was transferred from the 2nd tube to the 3rd tube, which also initially contained 150 ml of broth. This procedure was repeated until the required number of dilutions was reached. 150 ml of the mixture was taken from the last hole. Thus, the following dilutions were obtained: 1 : 1, 1 : 2, 1 : 4, 1 : 8, 1 : 16, 1 : 32, 1 : 64, 1 : 128, 1 : 256, 1 : 512, 1 : 1024, 1 : 2048, 1 : 4096, 1 : 8192, 1 : 16384, 1 : 32768, 1 : 65536, 1 : 131072, 1 : 262144, 1 : 524288, 1 : 1048576, and 1 : 2097152, corresponding to wells 1 to 23, with hole 24 being the control culture.

The concentration of the extract used in *in vitro* experiments was 66.2 *μ*g/ml.

**Figure 1:**
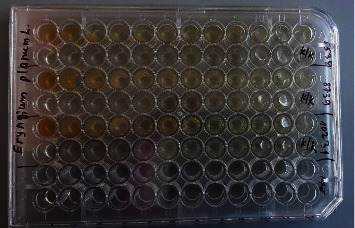
Setting antimicrobial activity in a 96-well plate.

After a series of dilutions, 20 *μ*l of test strains of microorganisms was added to all test tubes at a concentration of 1.5 × 10^6^ CFU/ml (Figure 1). All samples were incubated at 37 ± 1°C for 18–24 hours. After the incubation time, seeding was carried out on Petri dishes with Muller–Hinton nutrient medium to determine living cells. The presence of visible growth of microorganisms on the surface of a dense nutrient medium was taken into account in the results. The minimum bactericidal concentration (MBC) was considered the lowest concentration in a test tube, suppressing the growth of microorganisms.  Table 1 shows the labeling of Petri dishes according to dilutions.

#### 2.4.2. Determination of the Antimicrobial Activity of the Disk-Diffusion Method

The diffusion method was carried out by the borehole method. To perform this method, holes with a diameter of 6 mm were made with a sterile cylinder at a distance of 15–20 mm from the edge of the cup and from each other. The extract of *Eryngium planum* L. was added to the obtained wells, 80 ml each. Petri dishes were preinoculated with a suspension of test strains at a density of 1.5 × 10^8^ CFU/ml. Sterile cotton swabs were used for sowing and were immersed in a suspension of the microorganism, and then, these swabs were lightly pressed against the walls of the tube and hatched in three directions by turning the cup by 60°. As a reference discs with antibiotics were used (Ampicillin and Fluconazole).

After sowing, the cups were placed in a thermostat for incubation for 18–24 hours at 37°C for bacteria.

The results of the disk-diffusion method were taken into account by calculating the diameter of growth retardation/suppression zones with an accuracy of 1 mm.

## 3. Results and Discussion

### 3.1. Determination of the Compositional Breakdown of the CO_2_ Extract

**Figure 2:**
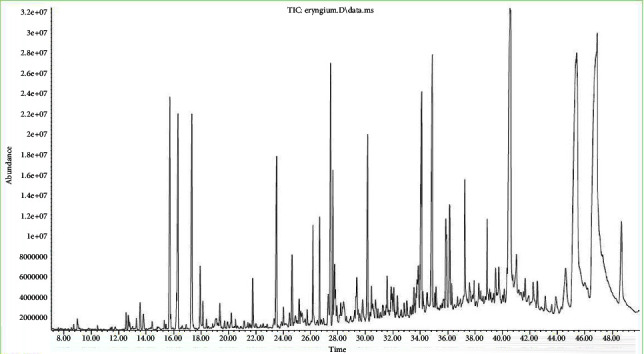
The analysis chromatogram of the *Eryngium planum* L. CO_2_ extract.

During the study, the phytochemical composition of the CO_2_ extract of the subterranean part of *Eryngium planum* L. was determined under subcritical conditions. 43 components were identified by chromatography-mass spectrometry, the main of which are *α*-linolenic acid, myristic acid, caryophyllene, spatulous, and other main identified compounds, and their percentage is shown in Table 2 and Figure 2.

The main components found in the CO_2_ extract under subcritical conditions were terpenes, sesquiterpenes such as caryophyllene (6.92%), spatulous (6.62%), and phytol (4.01%), and other compounds such as *α*-linolenic acid (8.30%) and myristic acid (6.40%). They indicate significant specific pharmacological activity, in particular antibacterial, anti-inflammatory, and antioxidant. We have studied the pharmacological activity of the main components by analyzing the work of foreign scientists. The nature of the chemical compound and its therapeutic activity are shown in Table 3.

It is worth noting that *β-*caryophyllene has antibacterial potential. In a study against *T strains. reuse, S. aureus.* and *E. coli*, with MIC values (minimum inhibitory concentrations), they were in the range from 3 to 14 microns [49]. Caryophyllene (14.9%) and spatulous (23.8%) exhibit antimicrobial activity against *Mycobacterium tuberculosis, Microspores gypsum, Trichophyton mentagrophytes,* and *Candida* [50].

Spatulous is one of the main compounds found in our study. It has antimicrobial, antiproliferative, and anti-inflammatory effects. In the study of *Eugenia calycina* leaf essential oil, spatulous showed antimicrobial activity against anaerobic bacteria such as *Prevelar nigricans* and *Porphyrins gingivitis* with an MIC (minimum inhibitory concentration) of 100 mcg/ml [51].

A significant contribution of our research consists in the identification and quantification of chemical compounds for the study of plant extracts.

### 3.2. Results of Antimicrobial Activity

When determining antimicrobial activity by the serial dilution method, antibacterial and fungicidal activity of the CO_2_ extract was found against the analyzed strains of *S. aureus, E. coli,* and *C. albicans* microorganisms.

To determine antimicrobial activity, the medium and test strains were used as a positive control to confirm growth for each test strain. For each test strain, suitable nutrient broth (Mueller–Hinton broth for bacterial testing or Sabouraud broth for fungal testing) without the test material was used as a negative control.

**Figure 3:**
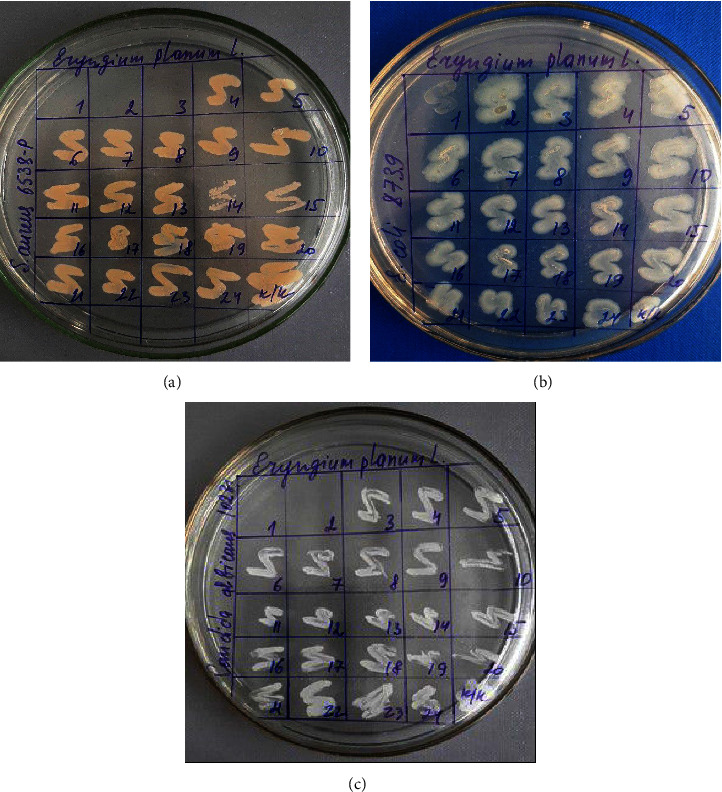
Results of the antimicrobial activity of *Eryngium planum*'s CO_2_ extract obtained by the serial dilution method. (a) *S. aureus*; (b) *E. coli*; (c) *C. albicans*.

The results were detected visually by the presence/absence of visible growth of microorganisms on the surface of a dense nutrient medium (Figure 3). The minimum bactericidal concentration (MBC) was considered the lowest concentration, which suppressed growth of microorganisms. The results of antibacterial activity of the carbon dioxide extract of *Eryngium planum* L. for the three pathogenic microorganisms are presented in Table 4.

It can be seen from the presented data that the test sample exhibits antimicrobial activity in relation to the test cultures under study. The CO_2_*Eryngium planum* L. extract was active against all tested strains. It has been experimentally shown that the extract of *Eryngium planum* L. when tested by serial dilutions, it has a bactericidal effect at a concentration of 8.3 mcg/mcl in relation to *Staphylococcus aureus* ATCC 6538-P; 33.1 mcg/mcl against *Escherichia coli* ATCC 8739; against yeast fungi 16.55 mcg/mcl*Candida albicans* ATCC 10231.

The test sample, carbon dioxide extract of *Eryngium planum* L. has antibacterial activity against *Staphylococcus aureus* ATCC 6538-P; against *Escherichia coli* ATCC 8739; and against yeast-like fungi *Candida albicans* ATCC 10231 by serial dilution in broth.

**Figure 4:**
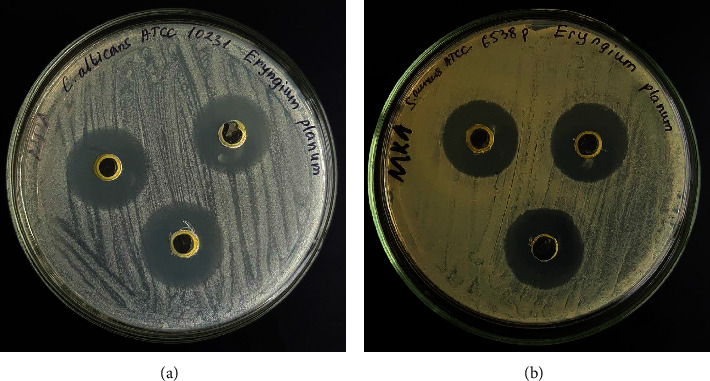
(a) Results of the antimicrobial activity of the ointment against the archival strain *C. albicans* ATCC 10231. (b) Results of the antimicrobial activity of the ointment against the museum strain *S. aureus* ATCC 6538-P.

The results of the study on the carbon dioxide extract of *Eryngium planum* L. by the disk-diffusion method are shown in Figure 4 and Table 5.

Ampicillin is a broad-spectrum, beta-lactam antibiotic active against Gram-positive and Gram-negative bacteria, and fluconazole is an antifungal medicine highly active against yeast-like fungi of the genus *Candida*. They were used as reference drugs, ampicillin 10 *μ*g/disc (HI media, India) and fluconazole 10 *μ*g/disc (HI media, India).

The test sample, a carbon dioxide extract of *Eryngium planum*, has antibacterial activity against *Staphylococcus aureus* ATCC 6538-P: its growth retardation zone is 18.67 ± 0.57 mm, which is 1.2 times more effective than control (the antibiotic ampicillin). Against the yeast-like fungus*Candida albicans* ATCC 10231, the growth retardation zone was 20.3 ± 0.57 mm, while the growth retardation zone of the antifungal drug fluconazole was 14.0 ± 0.0 mm.

Thus, the obtained results of the prototype indicate the effectiveness of the tested extract against Gram-positive bacteria and yeast-like fungi.

Antimicrobial activity of extracts has been described for some *Eryngium* species [6, 52–54]. Results of studies of antimicrobial activity of methanol extracts and fractions of *Eryngium planum*, *E. campestral*, and *E. maritime* by the microdilution method of broth showed that *maritime* and *planum* have the highest antimicrobial activity (MIC = 1-2 mg·ml^−1^) against *aureus*, followed by the saponin fraction of *planum* (MIC = 2.5 mg·ml^−1^). The inhibitory effect of methanol extracts of the roots of all the tested species (MIC = 12.5 mg·ml^−1^) and the *planum* cell suspension culture (MIC = 7.8 mg·ml^−1^) was found against *C. albicans* [52].

It is worth noting that the chemical composition of the essential oil plant depends on the type, climatic conditions, soil type, harvesting seasons, geographical region, and the extraction process used. Seasonal fluctuations are one of the main factors that affect the composition of essential oils [55, 56].

The composition of essential oil and antimicrobial activity of three other species belonging to the genus *Eryngium* (*creticum, campestris*, and *trifolium*), infusions of which are obtained from the aboveground and root parts. The antibacterial activity of essential oils was tested by the disk-diffusion method against nine clinical strains of methicillin-resistant*aureus* (MRSA). It has been demonstrated that the essential oil obtained from *trifolium*, which causes an inhibition zone in the range from 13 to 19 mm, has been demonstrated to be the most active species [6, 53]. Alcoholic extracts of *E. maritime* roots showed activity against *S. aureus* (MIC 2.5 mg·ml^−1^) [54].

## 4. Conclusion

To extract biologically active substances from the subterranean part of *Eryngium planum*, we have chosen carbon dioxide extraction technology, a technology for processing carbon dioxide (CO_2_) raw materials, which allows us to extract various substances in high concentrations. Carbon dioxide extraction technology is an effective and environmentally friendly way to isolate various biologically active substances contained in medicinal plant raw materials.

The sterility of extracts, low energy consumption, and relative cheapness of hardware today determine the advantages of subcritical carbon dioxide extraction over traditional methods of extracting substances. The parameters of the extraction process provide softer and gentler extraction of biologically active substances from plant raw materials, which makes it possible to obtain the substance in native form at the end of this process.

The chemical composition of the carbon dioxide extract of *Eryngium planum* obtained under subcritical conditions was studied for the first time. By chromatography-mass spectrometry, 43 components were identified, the main of which are *α*-linolenic acid, 8.30%; myristic acid, 6.40%; caryophyllene, 6.92%; and spatulous, 6.62%

The results of screening for antimicrobial activity showed that the CO_2_ extract of *Eryngium planum* exhibits antimicrobial activity against Gram-positive bacteria and yeast-like fungi, using the serial dilution method and the disk-diffusion method.

It has been experimentally found that the carbon dioxide extract of *Eryngium planum* has a high content of biologically active substances, which shows the relevance of the pharmacological study of the extract in order to use it as a medicinal product.

## Figures and Tables

**Table 1:** Labeling of Petri dishes according to dilutions.

Well 1	33.1
Well 2	16.55
Well 3	8.3
Well 4	4.1
Well 5	2.1
Well 6	
Well 7	1.03
Well 8	0.52
Well 9	0.26
Well 10	0.13
Well 11	0.06
Well 12	0.03
Well 13	0.016
Well 14	0.008
Well 15	0.004
Well 16	0.002
Well 17	0.001
Well 18	0.0005
Well 19	0.00025
Well 20	0.00013
Well 21	0.000063
Well 22	0.000032
Well 23	0.000016
Well 24	C

*Note*. C, culture control.

**Table 2:** The results of the chromatographic analysis of the *Eryngium planum* L. carbon dioxide extract.

S. no	Retention time (min)	Peak name	Identification probability (%)	Percentage (%)
1	8.6	Methyl sec-butyl disulphide	90	0.06
2	9.0	Octanal	94	0.33
3	10.5	1-Hexanol	88	0.09
4	11.4	2-Nonanone	85	0.05
5	11.5	Nonanal	79	0.07
6	11.7	Tetradecane	86	0.12
7	12.5	Disulfide, bis(1-methylpropyl)	85	0.45
8	12.8	1,2-Dithiolane	68	0.20
9	13.6	Cyclohexane, 1-ethenyl-1-methyl-2-(1-methylethenyl)-4-(1-methylethylidene)-	92	0.72
10	13.8	*α*-Copaene	92	0.53
11	14.4	*β*-Bourbonene	87	0.32
12	15.3	Tioxolone	64	0.28
13	15.7	Bicyclo[3.1.1]hept-2-en-4-ol, 2,6,6-trimethyl-, acetate	88	6.47
14	16.3	Caryophyllene	97	6.92
15	16.6	Nonadecane	73	0.10
16	17.3	*γ*-Elemene	93	6.12
17	17.9	Humulene	92	1.80
18	18.1	Globulol	72	0.81
19	19.4	Cyclohexane, 1-ethenyl-1-methyl-2-(1-methylethenyl)-4-(1-methylethylidene)-	91	0.84
20	19.9	Naphthalene, 1,2,3,4,4a,5,6,8a-octahydro-7-methyl-4-methylene-1-(1-methylethyl)-, (1*α*,4a*β*,8a*α*)-	87	0.30
21	21.2	2,4-Decadienal	79	0.26
22	23.3	Nonadecane	77	0.40
23	24.0	Heptanoic acid	91	0.59
24	24.7	Caryophyllene oxide	92	1.87
25	25.2	3,4,4-Trimethyl-3-(3-oxo-but-1-enyl)-bicyclo[4.1.0]heptan-2-one	80	0.83
26	25.8	12-Oxabicyclo[9.1.0]dodeca-3,7-diene, 1,5,5,8-tetramethyl-, [1R-(1R*∗*,3E,7E,11R*∗*)]-	83	0.51
27	26.7	Cyclohexanemethanol, 4-ethenyl-*α*,*α*,4-trimethyl-3-(1-methylethenyl)-, [1R-(1*α*,3*α*,4*β*)]-	95	2.58
28	27.5	Spathulenol	93	6.62
29	27.9	Cadinol	78	0.73
30	30.5	Aromadendrene oxide	75	2.49
31	33.8	Dodecanoic acid	90	4.13
32	34.9	9,12-Octadecadienoic acid, ethyl ester	91	12.41
33	36.1	Phytol	87	4.01
34	37.3	Tetradecanoic acid	86	6.40
35	37.6	2-Tert-butylcyclohexyl	85	2.21
36	37.9	Heptacosane	84	1.84
37	38.9	Platambin	75	5.24
38	40.0	Palmitoleic acid	86	1.23
39	41.7	4,8,12,16-Tetramethylheptadecan-4-olide	86	2.01
40	42.6	Falcarinol	85	1.76
41	43.9	Squalene	88	1.60
42	44.6	Octadecanoic acid	90	5.37
43	48.7	9,12,15-Octadecatrienoic acid	94	8.30

**Table 3:** Nature of the compounds present in the CO_2_ extract of *Eryngium planum* L.

S. no	Compound	Compound nature	Therapeutic activity	PubChem CID
1	Octanal	Aldehyde	Allelopathic activity [33]	454
2	*α*-Copaene	Sesquiterpene	Antioxidant activity [34], antigenotoxic, anticytotoxic, and anticytogenetic effects [35]	70678558
3	*β*-Bourbonene	Sesquiterpenoid	Anticancer [36]	62566
4	Tioxolone	Organic compound	Antipsoriatic and antibacterial properties, anti-inflammatory effects [37]	72139
5	Caryophyllene	Terpene	Antibacterial, anti-inflammatory effects [38]	5281515
6	*γ*-Elemene	Sesquiterpene	Antiproliferative effect [39]	6432312
7	Humulene	Sesquiterpene	Anti-inflammatory, antibacterial action effects [40]	5281520
8	Globulol	Sesquiterpene	Antimicrobial effect [41]	101716
9	Caryophyllene oxide	Terpene	Antibacterial, anti-inflammatory effects [38]	1742210
10	Spathulenol	Tricyclic sesquiterpenoid	Antioxidant, anti-inflammatory, antiproliferative and antimicrobial effects [42]	92231
11	9,12-Octadecadienoic acid, ethyl ester	Linolenic acid	Insecticidal, hepatoprotective, antihistamine, hypocholesterolemic, and antieczematic effects [43].	5365672
12	Phytol	Diterpene	Regenerating, toning, and antimicrobial effects [43]	5280435
13	Tetradecanoic acid	Carboxylic acid	Helps restore the protective properties of the skin and possesses excellent sliding and lubricating properties [43, 44]	11005
14	Platambin	Sesquiterpene	Antibacterial effect [45]	613194
15	Falcarinol	Fatty alcohol	Antitumor activity, antibacterial effect [46]	5281149
16	Squalene	Triterpene	Antioxidant and antitumor activities [47]	638072
17	Octadecanoic acid	Linolenic acid	Antibacterial and antifungal [48]	5282750
18	9,12,15-Octadecatrienoic acid	*α*-linolenic acid	Analgesic, allergenic, antibacterial, anti-inflammatory, and antioxidant [43, 48]	860

**Table 4:** Results of the antimicrobial activity of the extract of *Eryngium planum* L. obtained by the serial dilution method.

Test strains	Minimum bactericidal dilution of the extract of *Eryngium planum*L. (*μ*g/*μ*l)
	Bactericidal effect
*Staphylococcus aureus* ATCC 6538-р	8.3
*Escherichia coli* ATCC 8739	33.1
*Candida albicans* ATCC 10231	16.55

**Table 5:** Results of the antimicrobial activity of the *Eryngium planum* extract obtained using the disk-diffusion method.

Test strain	Growth exhibition zone *М* ± Std (mm)	
	*Eryngium planum* L. extract	Control
*Staphylococcus aureus* ATCC 6538-P	18.67 ± 0.57	Ampicillin 15.6 ± 0.57
*Candida albicans* ATCC 10231	20.3 ± 0.57	Fluconazole 14.0 ± 0.0

## Data Availability

The data used to support the findings of this study are included within the article.
